# Level-Aware Residual Mixup for Defect Classification in Material Extrusion with Limited Print Jobs

**DOI:** 10.3390/ma19173769

**Published:** 2026-09-04

**Authors:** Yeonggyeom Kim, Sangho Lee

**Affiliations:** 1 School of Industrial and Systems Engineering, Gyeongsang National University, 501 Jinju-daero, Jinju 52828, Republic of Korea; kyk090830@gnu.ac.kr; 2 Graduate School of Aerospace and Defense Convergence, Gyeongsang National University, 501 Jinju-daero, Jinju 52828, Republic of Korea

**Keywords:** additive manufacturing, material extrusion, in-situ process monitoring, defect classification, data augmentation

## Abstract

Reliable in-situ defect classification is essential for ensuring the mechanical performance of parts produced by material extrusion. However, machine learning and deep learning models often suffer from poor generalization because training datasets typically consist of many correlated signal segments generated from only a limited number of independent print jobs. To address this problem, we analyze how job-specific characteristics are distributed across signal components and find that they are concentrated primarily in the signal level rather than in the residual component. Motivated by this observation, we propose Level-Aware Residual Mixup(LARM), a data augmentation method that separately interpolates the level and residual components of sensor signals. LARM preserves realistic job-level characteristics by restricting level interpolation to values observed in real print jobs while allowing flexible mixing of residual components within the same defect class. We evaluate LARM against representative augmentation methods across diverse classification models under a realistic job-level leave-one-group-out cross-validation protocol. Experimental results demonstrate that LARM achieves better generalization than both training without augmentation and representative augmentation methods.

## 1. Introduction

Additive manufacturing has been increasingly adopted across aerospace, biomedical, and consumer product industries owing to its ability to fabricate geometrically complex and customized components with high design flexibility and on-demand production [1]. Among various additive manufacturing technologies, polymer-based processes, particularly material extrusion (MEX), have been widely used due to their accessibility, relatively low cost, and material versatility [2]. Despite these advantages, the layer-by-layer deposition mechanism of MEX introduces process-dependent variability and anisotropic material behavior, making it challenging to achieve consistent mechanical performance [3,4].

To ensure reliable mechanical performance in MEX, process parameters such as extrusion temperature, printing speed, and nozzle and filament conditions should remain within their intended operating ranges, as deviations can lead to defects that degrade the structural integrity of the final part [4,5,6]. Thus, detecting such deviations during fabrication is essential for timely corrective actions before defective components are produced. In this context, classifying defects from in-situ monitoring signals provides a preventive strategy that complements conventional post-process inspection and mechanical testing [7].

In general, defects arise from complex interactions among multiple process parameters and are reflected in sensor signals through subtle temporal patterns, fluctuations, and cross-channel dependencies [2,7]. Thus, reliable defect classification depends on effectively capturing these complex signal characteristics, motivating the increasing adoption of data-driven approaches, particularly machine learning and deep learning [8,9,10]. For example, Westphal and Seitz [11] explored the potential of these approaches using a range of deep learning architectures, including a multilayer perceptron (MLP), a one-dimensional convolutional neural network (CNN), and a long short-term memory (LSTM), achieving high classification accuracy in distinguishing normal and defective process conditions from environmental sensor recordings collected during MEX. In addition, Khusheef et al. [12] introduced a deep learning framework that fuses signals from multiple sensors for process monitoring in MEX.

However, achieving such promising performance depends on the availability of sufficiently diverse training data, which is difficult to obtain in practical MEX settings [8]. Although each print job produces long, densely sampled sensor recordings, the number of independent print jobs available for training is often limited due to the time, material, and operational costs associated with conducting experiments. Consequently, a large number of training samples may originate from only a small set of print jobs, resulting in a repeated-measures structure in which samples from the same job are highly correlated [13]. Under such conditions, models are likely to learn job-specific characteristics that are unrelated to the underlying defect classes, failing to generalize to unseen print jobs despite achieving strong performance on samples from known jobs, a characteristic symptom of overfitting [14].

Several strategies can be employed to mitigate the overfitting caused by the repeated-measures structure. Collecting additional print jobs would directly increase the number of independent jobs available for training, but acquiring such data incurs substantial time, material, and operational costs. Moreover, synthetic sensor recordings may be generated through process simulation, but this approach requires a validated physical model that is often unavailable in practice [15]. In contrast, data augmentation is a practical and widely adopted strategy for improving model generalization under limited training data [16,17,18]. By generating diverse training examples from the available print jobs, data augmentation can improve model generalization without requiring additional experiments, additional independent print jobs, or an explicit process model.

A wide range of augmentation strategies has been proposed for time-series signals, including direct signal perturbations and sample-mixing strategies [16,19]. Despite their effectiveness, most existing augmentation strategies are designed to increase sample diversity without explicitly considering the repeated-measures structure of the data. Consequently, they generally operate without distinguishing between characteristics that are specific to individual print jobs and those that are shared across print jobs. This distinction may be important when job-specific characteristics are distributed unevenly across different signal components, as augmentation without accounting for such differences may limit generalization to unseen print jobs.

This limitation also applies to mixup [20], which has been widely adopted owing to its simplicity and effectiveness in improving generalization [21]. Standard mixup generates synthetic samples by interpolating pairs of training examples using a common mixing ratio for the entire signal, without considering potential differences in job-specific information across signal components. Thus, understanding how job-specific characteristics are distributed across signal components is important for designing augmentation strategies that account for the repeated-measures structure.

To examine how job-specific characteristics are distributed across signal components, we quantify the extent to which different signal components vary between print jobs rather than within them. This analysis indicates that job-specific characteristics are predominantly associated with the level component of a sensor signal, representing its overall magnitude or offset, rather than with the residual component, which reflects temporal fluctuations around that level. To further examine how this finding relates to augmentation performance, we compare a diverse set of representative augmentation strategies across a range of machine learning and deep learning models for defect classification in MEX. The comparison shows that augmentation strategies that alter the signal level tend to provide limited benefits.

Motivated by these findings, we propose Level-Aware Residual Mixup (LARM), a novel augmentation method that extends mixup by separately mixing the level and residual components of a sensor signal. While standard mixup treats all signal components equally during interpolation, its linear interpolation formulation naturally allows different signal components to be mixed independently. Leveraging this property, LARM freely mixes the residual component between samples from the same defect class while restricting level interpolation to values observed in real print jobs. Experimental results show that LARM consistently improves generalization performance across a diverse set of machine learning and deep learning models, outperforming both conventional augmentation strategies and training without augmentation.

The main contributions of this study are as follows:

- We characterize how job-specific characteristics are distributed across the level and residual components of sensor signals and show that they are predominantly associated with the level component rather than the residual component.
- We propose a novel data augmentation method, LARM, that separately handles the level and residual components of sensor signals by freely mixing residual components while constraining level interpolation to values observed in real print jobs.
- Experimental results demonstrate that LARM consistently improves generalization performance across various machine learning and deep learning models, outperforming both training without augmentation and conventional augmentation strategies.

## 2. Methods

**Figure 1:**
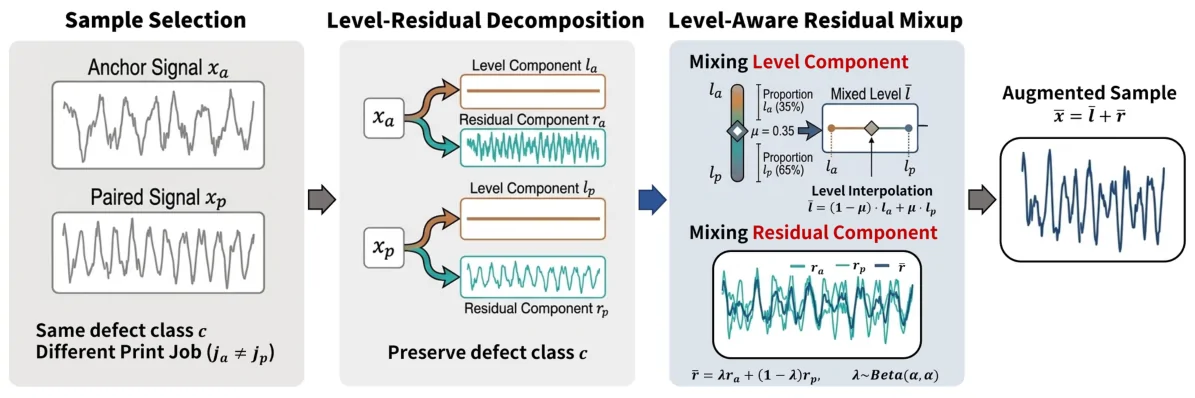
Overview of the proposed method.

The proposed method is based on the observation that job-specific characteristics are not distributed uniformly across a sensor signal. To exploit this property, we decompose each signal into a level component and a residual component and handle the two components differently during augmentation. The following subsections formally define the decomposition and present our method, LARM, in detail. An overview of the proposed method is illustrated in Figure 1.

### 2.1. Level-Residual Decomposition

Let $D={\left\{x_{i}\right\}}_{i=1}^{N}$ be a set of fixed-length segments of multichannel in-situ sensor recordings acquired across multiple MEX print jobs, where each segment $x_{i}\in R^{T\times C}$ consists of *T* time steps and *C* sensor channels. In this subsection, we omit the sample index and denote a representative segment simply by $x\in R^{T\times C}$, where $x_{t,c}$ denotes the observation at time step *t* in channel *c*.

To separate the overall magnitude of a signal from its temporal fluctuations [22], we decompose *x* into two additive components: a level component $l\in R^{C}$ and a residual component $r\in R^{T\times C}$.

The level component captures the average magnitude of each sensor channel while discarding short-term temporal fluctuations. Under the steady-state printing conditions considered in this study, sensor measurements are expected to fluctuate around a stable operating point without exhibiting systematic drift within a segment [23]. Accordingly, we represent the level by the channel-wise mean rather than a linear or higher-order trend, which would unnecessarily increase model complexity and absorb variations intended to be captured by the residual component. Specifically, for each channel *c*, the level component is defined as

(1) $$l_{c}=\frac{1}{T}\sum_{t=1}^{T}x_{t,c},$$

where $l_{c}$ represents the average magnitude, or offset, around which observations in channel *c* fluctuate within the segment. Since one level value is computed for each channel, the level component is represented as a *C*-dimensional vector.

The residual component captures the temporal fluctuations that remain after removing the level component and is defined as

(2) $$r_{t,c}=x_{t,c}-l_{c},$$

where $r_{t,c}$ represents deviations from the channel-wise average magnitude, preserving local temporal patterns while removing the influence of the channel-wise offset. The resulting residual component has the same dimensionality as the original signal. Rearranging the definition of the residual shows that the decomposition is lossless, as the original signal can be recovered exactly as $x_{t,c}=l_{c}+r_{t,c}$. Thus, the level and residual components provide an alternative representation of the original signal without discarding any information.

This decomposition is motivated by the hypothesis that job-specific characteristics are not distributed uniformly across a sensor signal. Characteristics that vary across print jobs while remaining relatively stable within an individual print job, such as ambient environmental conditions, sensor calibration, machine-specific characteristics, and fixed process settings, are expected to appear primarily as shifts in the average magnitude of sensor measurements and therefore to be captured by the level component [24]. In contrast, temporal fluctuations around the level are less likely to reflect differences between print jobs and are therefore expected to be represented primarily by the residual component [25]. The validity of this hypothesis is examined empirically in Section 3.2.1 and Section 3.2.2. Motivated by this hypothesis, the following subsection introduces an augmentation strategy based on mixup that handles the level and residual components differently.

### 2.2. Level-Aware Residual Mixup

Motivated by the hypothesis that job-specific characteristics are concentrated primarily in the level component, we extend mixup by treating the level and residual components differently during augmentation. Since mixup generates synthetic samples through linear interpolation, the interpolation process can be applied independently to any additive decomposition of a signal. Thus, we first decompose each signal segment into its level and residual components and then apply separate mixing strategies to the two components. Specifically, the level component is mixed only between levels observed in real print jobs, whereas the residual component is mixed freely to retain the diversity benefits of mixup. The mixed level and residual components are then recombined to construct an augmented signal.

Let $y_{i}\in \left\{1,\ldots ,K\right\}$ and $j_{i}$ denote the defect class and print job associated with segment $x_{i}\in D$, respectively. To generate an augmented sample, we first select a defect class *y* uniformly from {1,…,K}. Then, an anchor sample $x_{a}$ is drawn uniformly from the subset of D with label *y*, and a paired sample $x_{p}$ is selected from the same class such that $j_{p}\neq j_{a}$. That is, $x_{p}$ is required to originate from a print job different from that of $x_{a}$. If no such sample exists, $x_{p}$ is instead drawn from the same print job as $x_{a}$. Restricting both samples to the same defect class preserves label consistency, allowing the augmented sample to inherit the selected class label. This design further encourages the classifier to learn class-relevant patterns that generalize across print jobs rather than overfitting to job-specific characteristics.

After selecting the anchor and paired samples, $x_{a}$ and $x_{p}$, LARM first decomposes each signal into its level and residual components, yielding $\left(l_{a},r_{a}\right)$ and $\left(l_{p},r_{p}\right)$ (see Section 2.1). The two components are then mixed separately using different mixing strategies.

Even within the same defect class, residual patterns may vary across print jobs. Since the residual component is expected to contain relatively little job-specific information, residual components from different print jobs can be freely combined to generate additional within-class temporal patterns, thereby enriching the diversity of within-class temporal dynamics. Accordingly, the residual components are combined as

(3) $$\bar{r}=\lambda r_{a}+\left(1-\lambda \right)r_{p},\;\lambda ∼\mathrm{Beta}\left(\alpha ,\alpha \right),$$

where λ is independently sampled for each augmented sample from a symmetric Beta distribution, following the standard mixup formulation [20]. The parameter α controls the degree of residual interpolation, with smaller values favoring mixtures closer to one of the two residual components and larger values producing more balanced combinations.

Unlike the residual component, the level component is hypothesized to carry a substantial portion of the variation that distinguishes one print job from another. Thus, unconstrained modification of the level component may produce levels that are not representative of those observed in real print jobs. To preserve plausible signal levels while still allowing interpolation between those observed in real print jobs, the level components are mixed as

(4) $$\bar{l}=\left(1-\mu \right)l_{a}+\mu l_{p},\;\mu \in \left[0,1\right],$$

where μ is the level-mixing hyperparameter. Unlike the residual mixing ratio λ, which is randomly sampled to introduce diverse residual variations, μ is fixed to explicitly control the degree of level mixing. Since $\bar{l}$ is constructed as a convex combination of two levels observed in real print jobs, its value always lies between levels observed in the training data. The parameter μ determines where $\bar{l}$ lies within this interval. For example, when μ=0, the level is inherited entirely from the anchor sample, whereas μ=1 replaces it completely with the level of the paired sample.

Finally, the augmented sample is generated by recombining the mixed level and residual components as follows:

(5) $${\bar{x}}_{t,c}={\bar{l}}_{c}+{\bar{r}}_{t,c}.$$

The resulting sample inherits the class label *y* because both source samples belong to the same defect class. Note that the decomposition is used only during augmentation; after recombination, the augmented sample has the same form as the original signal and is used directly as input to the classifier.

## 3. Experiments

To investigate how job-specific characteristics are distributed across the level and residual components of MEX sensor signals and to evaluate the effectiveness of the proposed method, we conduct a comprehensive set of experiments.

### 3.1. Experimental Setup

Here, we briefly describe the experimental settings, including the dataset, classification models, evaluation protocol, augmentation strategies, and implementation details.

#### 3.1.1. Dataset

We used the FDM (Fused Deposition Modeling) Environmental Sensor Dataset introduced by Westphal and Seitz [11] and publicly available in the Mendeley Data repository (https://doi.org/10.17632/pprxj2yfby.1; accessed on 29 August 2026), which was collected to support sensor-based quality assurance in MEX. An environmental sensor (BME680, Bosch Sensortec GmbH, Reutlingen, Germany) mounted near the print nozzle recorded four channels—gas resistance, relative humidity, air pressure, and ambient temperature—at a sampling rate of 2 Hz throughout each print job.

Print jobs were carried out under one normal and three defective process conditions generated by modifying specific process parameters. The Normal condition represents a typical continuous-production setting, using a worn nozzle and new PLA+ filament (Filamentworld, Neu-Ulm, Germany) at the recommended printing temperature of 210 °C. Defect_01 replaced the new filament with filament that had been stored openly for at least 12 months. Defect_02 replaced the worn nozzle with a new, unused nozzle. Defect_03 combined a new nozzle with an elevated printing temperature of 240 °C.

Each print job’s continuous recording was partitioned into non-overlapping segments of T=500 samples (250 s at 2 Hz), yielding 40 segments per job. The dataset comprises 19 independent print jobs, ten under the Normal condition and three each under Defect_01, Defect_02, and Defect_03, for a total of N=760 segments. This results in a moderate class imbalance, with the Normal class accounting for 400 of the 760 segments. Although the dataset contains 760 signal segments, they originate from only 19 independent print jobs, resulting in a repeated-measures structure in which segments from the same print job are likely to be correlated.

#### 3.1.2. Classification Models

To evaluate the effectiveness of LARM across diverse modeling paradigms, we considered eight classification models spanning classical machine learning and deep learning approaches. The deep learning models were selected to represent three widely adopted architectures for time-series modeling: convolutional, recurrent, and attention-based networks [18].

For the classical machine learning models, we considered logistic regression, random forest [26], and a support vector machine with a radial basis function kernel [27]. As commonly adopted in sensor-based condition monitoring [28,29], these models were trained on handcrafted statistical features. Specifically, we computed six time-domain statistical features [30], namely the mean, standard deviation, minimum, maximum, median, and linear trend slope, for each of the four sensor channels, yielding a 24-dimensional feature vector for each segment.

In contrast to the machine learning models, the deep learning models operated directly on raw signals. Recurrent architectures were represented by LSTM [31] and gated recurrent unit (GRU) [32], each consisting of two recurrent layers with 64 hidden units and a dropout rate of 0.2. Convolutional architectures were represented by a deep CNN with wide first-layer kernels (WDCNN) [33], comprising an initial convolution with a kernel size of 16 and stride 4, followed by three convolutional blocks with 32, 64, and 64 filters, each with a kernel size of 3. Finally, attention-based architectures were represented by a Transformer [34] and PatchTST [35], both employing two-layer Transformer encoders with a model dimension of 64, four attention heads, a feedforward dimension of 128, and a dropout rate of 0.1. While the Transformer processes all channels jointly at each time step, PatchTST operates on non-overlapping patches of 25 time steps and performs channel-independent encoding.

#### 3.1.3. Evaluation Protocol

To evaluate generalization to unseen print jobs, we adopted a leave-one-group-out cross-validation protocol [13,36,37], in which print jobs were treated as groups. Unlike a conventional random train–test split, this protocol prevents segments from the same print job from appearing in both the training and test sets. Since segments from the same job may share job-specific characteristics, random splitting can lead to overly optimistic performance estimates by allowing the model to exploit print-job information rather than learn defect-related patterns [14,38]. In contrast, leave-one-group-out cross-validation provides a more realistic assessment of generalization by evaluating the model on entirely unseen jobs. The adopted protocol created one fold per print job, with each fold corresponding to a different held-out job. Since the dataset contained 19 print jobs, a total of 19 folds were generated. In each fold, all segments from the held-out job were reserved for testing, while the classifier was trained on segments from the remaining 18 jobs.

#### 3.1.4. Augmentation Strategies

We compared LARM with a diverse set of representative time-series augmentation strategies. Specifically, we included jitter, scaling, time warping, and cutout, which are representative transformation-based approaches [16,39]. We also included dynamic time warping (DTW) barycenter averaging (DBA) [40], frequency masking [41], the synthetic minority over-sampling technique (SMOTE) [42], and standard mixup [20].

The compared augmentation strategies are summarized as follows:

- Jitter: Adds Gaussian noise scaled to the standard deviation of each channel.
- Scaling: Multiplies each channel by a randomly sampled scaling factor.
- Time Warping: Applies a smooth random warping curve to nonlinearly distort the time axis.
- Cutout: Replaces a randomly selected contiguous segment with the channel-wise mean.
- DBA: Combines two same-class segments through DTW alignment followed by barycenter averaging of the original signals.
- Frequency Masking: Removes a randomly selected frequency band from each channel before reconstructing the signal in the time domain.
- SMOTE: Generates a synthetic segment by interpolating between a sample and one of its *k* nearest same-class neighbors.
- Mixup: Combines two same-class segments, sampled either across or within print jobs, by linearly interpolating the original signals without decomposition.

#### 3.1.5. Implementation Details

The machine learning models were implemented using scikit-learn (version 1.8.0) [37]. For logistic regression and SVM, extracted features were standardized using the mean and standard deviation computed from the training set only, whereas random forest was trained on the original features. The deep learning models were implemented using PyTorch (version 2.5.1) [43] and trained using the Adam optimizer implemented in PyTorch [44]. Each input channel was standardized using statistics computed from the training set only.

For all augmentation strategies, the number of synthetic samples was set equal to the size of the original training set, and the generated samples were appended to the original samples. Jitter added channel-wise Gaussian noise, while scaling applied a random multiplicative factor to each channel. Time warping applied a smooth temporal distortion, cutout replaced a contiguous segment with the channel-wise mean, and frequency masking removed a randomly selected frequency band. SMOTE generated samples by interpolating between each sample and a nearest same-class neighbor, whereas DBA performed barycenter averaging after DTW-based alignment. Standard mixup followed the conventional interpolation formulation, while LARM followed the procedure described in Section 2.2.

All experiments were repeated across five random seeds, and the mean and standard deviation were reported. Detailed implementation settings and the computational environment are summarized in Table 1.

### 3.2. Experimental Results

We first analyze the extent to which job-specific characteristics are captured by the level and residual components. Then, we compare representative augmentation strategies across various classification models and finally present additional analyses and ablation studies of LARM.

#### 3.2.1. Job-Specific Information in Level and Residual Components

To investigate whether job-specific characteristics are concentrated in the level or residual component, we quantify the extent to which signal variation is explained by print job identity using the intraclass correlation coefficient (ICC) [13,45]. The ICC measures the proportion of total variance attributable to between-group differences. In our setting, the groups correspond to print jobs. An ICC value close to 1 indicates that samples from the same print job are highly similar to one another, suggesting that the corresponding signal component contains substantial job-specific information. Conversely, an ICC value close to 0 indicates little variation associated with print job identity.

ICC is computed using a one-way random-effects analysis of variance, treating print jobs as the grouping factor. Since print jobs are nested within defect classes in this dataset, for the ICC analysis, each observation is first centered by its corresponding class mean so that the resulting ICC reflects variation attributable to print job identity rather than defect class differences. For the level component, the ICC is computed directly from the centered level values. For the residual component, the ICC is computed independently at each time step from the centered residual values and then averaged across all time steps. Following the one-way random-effects formulation of McGraw and Wong [45], the ICC is computed as

(6) $$\mathrm{ICC}=\frac{MS_{B}-MS_{W}}{MS_{B}+\left(n_{0}-1\right)MS_{W}},$$

where $MS_{B}$ and $MS_{W}$ denote the between-job and within-job mean squares, respectively, and $n_{0}$ denotes a correction term that accounts for unequal print-job sizes. Note that negative ICC estimates are truncated to zero, as such values arise from sampling variability and do not represent meaningful group effects.

Table 2 summarizes the ICC values for the four sensor channels. The level component exhibits consistently high ICC values across all channels, ranging from 0.307 (temperature) to 0.998 (pressure), with an average of 0.686. This indicates that a substantial proportion of the variation in the signal level, beyond that explained by defect class, is attributable to differences between print jobs. In contrast, the residual component exhibits ICC values close to zero across all channels, ranging from 0.000 to 0.009, with an average of 0.003. These results suggest that residual fluctuations contain little systematic information related to print job identity.

These findings support the hypothesis that job-specific characteristics are concentrated primarily in the level component rather than the residual component. They also motivate the design of LARM, which preserves realistic level characteristics by interpolating only between levels observed in real print jobs while allowing flexible mixing of residual variations.

#### 3.2.2. Comparative Evaluation of Augmentation Strategies

To examine the practical implications of the preceding ICC analysis, we compare a baseline without augmentation, eight representative augmentation methods, and the proposed method, LARM, across all eight classification models.

Table 3 reports the mean and standard deviation of classification accuracy for each augmentation-model combination. To assess whether the performance improvements achieved by LARM are statistically significant, we perform paired *t*-tests comparing LARM with the baseline across five random seeds. The superscripts * and ** indicate statistical significance at the p<0.10 and p<0.05 levels, respectively. The corresponding *p*-values are reported in the last row of Table 3.

As shown in Table 3, the effectiveness of existing augmentation strategies varies substantially across classification models. While some strategies improve performance for particular architectures, their effects are often inconsistent and can even degrade performance in other cases. These performance differences can be understood by considering how each augmentation technique affects the level and residual components.

Jitter and time warping primarily modify residual variations without altering the signal level. Similarly, cutout preserves the overall signal level by replacing removed segments with the window mean, although it substantially perturbs local residual patterns. As a result, these strategies generally preserve realistic level characteristics and often lead to performance improvements.

In contrast, scaling multiplies the entire signal, including its level, by an unconstrained random factor and can therefore shift the level beyond the range observed in real print jobs. To directly quantify this level distortion, we analyzed the levels generated by scaling. For each fold, the level range, mean, and standard deviation were calculated using only the training print jobs. We measured the fraction of augmented samples whose levels fell outside the observed training range and the mean absolute z-score of these out-of-range samples, which indicates their average standardized deviation from the training mean. As shown in Table 4, scaling generated out-of-range levels for all four channels, with particularly high fractions for pressure (0.907±0.005) and temperature (0.395±0.009). The out-of-range pressure levels also exhibited a mean absolute z-score of 11.332±0.315, indicating substantial deviations from the levels observed in the training print jobs. Consistent with this level distortion, scaling degrades performance for all eight models, reducing accuracy by 0.074 to 0.237 relative to the baseline.

Meanwhile, frequency masking exhibits pronounced model dependence. Since its effect on the signal level depends on the randomly selected frequency band, the resulting level characteristics are not explicitly controlled. Consequently, it improves accuracy for recurrent architectures (LSTM: +0.046; GRU: +0.050) while degrading performance for logistic regression, SVM, and WDCNN by up to 0.088. In addition, DBA, SMOTE, and standard mixup do not explicitly preserve realistic level characteristics. By operating on the signal without distinguishing between level and residual information, these methods may alter the level component in ways that do not correspond to those observed in real print jobs, resulting in inconsistent effectiveness across architectures.

These observations are consistent with the ICC analysis in Table 2, which shows that the level component contains considerably more job-specific information than the residual component. This suggests that preserving realistic level characteristics is important for effective augmentation under the job-level evaluation protocol.

The performance of LARM further supports this interpretation. Specifically, by constraining the level component while independently mixing residual variations, LARM explicitly accounts for the distinct roles of the level and residual components during augmentation. Empirically, LARM achieves the highest accuracy among all compared augmentation strategies for every classification model. These improvements are statistically significant for seven models at the p<0.10 level and for five models at the p<0.05 level.

#### 3.2.3. Comparison with Standard Mixup

**Figure 2:**
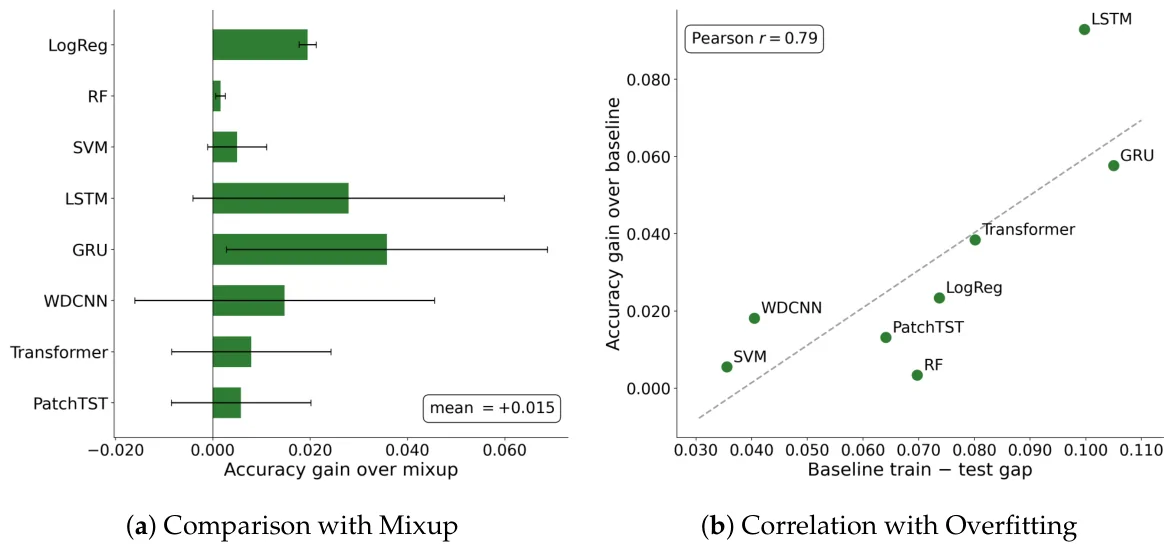
(**a**) Accuracy gain achieved by LARM compared to standard mixup. (**b**) Relationship between the baseline train–test accuracy gap and the accuracy gain achieved by LARM.

LARM extends standard mixup by explicitly distinguishing between the level and residual components during interpolation. To examine the contribution of the component-aware mixing strategy more directly, Figure 2a summarizes the accuracy gain of LARM over standard mixup under the leave-one-group-out evaluation protocol. Although the magnitude of improvement varies across models, the gain remains positive in all eight cases, indicating that the benefit of LARM is not confined to a particular modeling paradigm.

These results provide direct empirical evidence for the effectiveness of the component-aware extension to standard mixup. Standard mixup interpolates the entire signal without explicitly constraining the level of the augmented signal, allowing level information to be modified in ways that may not reflect realistic print-job characteristics. In contrast, LARM constrains the level component while independently mixing residual variations, thereby preserving more realistic job-specific characteristics during interpolation. The uniformly positive gains observed across all eight models indicate that explicitly accounting for the distinct roles of the level and residual components, while preserving realistic level characteristics, is beneficial for augmenting MEX data with a repeated-measures structure and a limited number of independent print jobs.

#### 3.2.4. Ablation Study of Level and Residual Mixing

To examine the contribution of separately controlling the level and residual components, we conducted an ablation analysis with four alternative mixing conditions in addition to LARM. In level-only mixing, the level components of two same-class samples from different print jobs were interpolated using λ∼Beta(0.4,0.4), while the residual component of the anchor sample was retained. Residual-only mixing corresponds to LARM with μ=0, where only the residual component is mixed. Standard mixup applies the same mixing ratio to the entire signal and therefore couples the mixing of the level and residual components. No mixing corresponds to the original training data without augmentation.

As shown in Table 5, neither level-only nor residual-only mixing consistently outperforms the other across classifiers, indicating that the relative contribution of the two components depends on the classification model. Standard mixup also improves accuracy for some classifiers but does not consistently outperform the component-specific variants. In comparison, LARM achieves the highest accuracy across most classifiers. These results indicate that the performance improvement of LARM cannot be attributed solely to mixing either the level or residual component and support the benefit of separately controlling the mixing of the two components.

#### 3.2.5. Relationship Between Baseline Overfitting and Augmentation Benefit

Since LARM is intended to mitigate overfitting caused by the repeated-measures structure and the limited number of independent print jobs, we investigate whether models that are more prone to overfitting benefit more from LARM. To this end, we examine the relationship between baseline overfitting and the performance gain achieved by LARM.

For each model, we quantify baseline overfitting as the difference between training accuracy and test accuracy under the leave-one-group-out evaluation protocol, without augmentation. We then compute the correlation between this baseline train–test gap and the accuracy improvement achieved by LARM over the baseline.

Figure 2b shows that models exhibiting larger baseline train–test gaps generally obtain greater performance gains from LARM (Pearson r=0.740). For example, LSTM and GRU exhibit the largest baseline gaps (0.092 and 0.101, respectively) and also achieve the largest improvements from LARM (0.093 and 0.058, respectively). Conversely, SVM and WDCNN exhibit relatively small baseline gaps (0.036 and 0.040, respectively) and correspondingly modest improvements (0.006 and 0.017, respectively). The observed relationship between baseline overfitting and the effectiveness of LARM is consistent with the motivation of LARM and suggests that it is particularly effective for classifiers that are more susceptible to overfitting in MEX settings.

#### 3.2.6. Robustness to Limited Print Jobs

In practical MEX applications, only a limited number of completed print jobs may be available when an in-situ defect classifier is first deployed. Although LARM is designed to mitigate the challenges arising from this limited availability of independent print jobs, its effectiveness may still depend on the amount of training data available. To examine its robustness under more severe data scarcity, we evaluate its performance using a substantially reduced training set.

For each leave-one-group-out fold, up to 18 training print jobs are available after excluding the test job. To reduce sampling variability, we randomly generate three different subsets of K=9 training jobs for each fold. Both the baseline and LARM are trained using only the selected training jobs. We then compare the accuracy gain achieved by LARM over the baseline with the corresponding gain obtained using the full training set.

**Figure 3:**
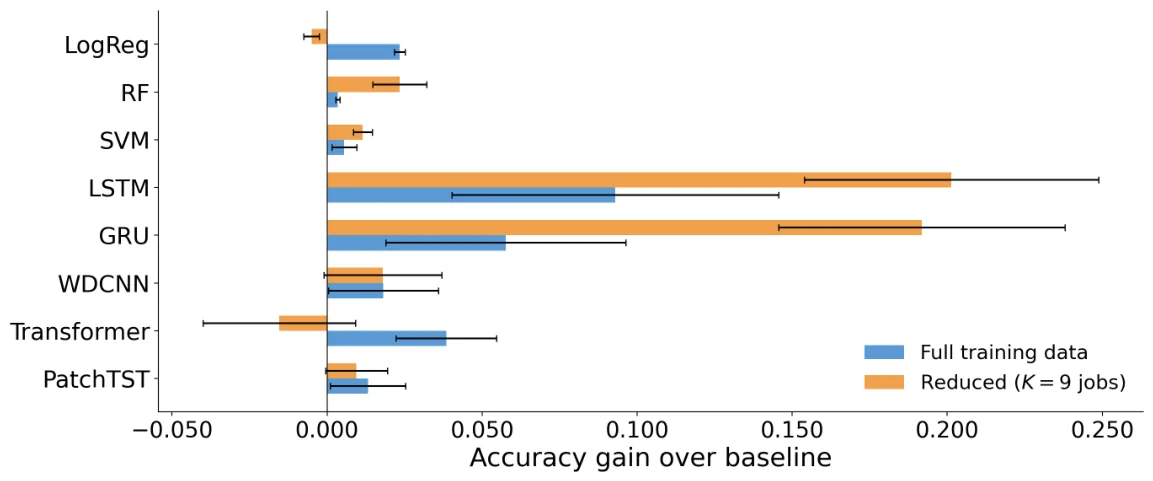
Accuracy gain of LARM over the baseline for each classification model under the full-data and reduced-data (K=9 print jobs) settings.

As shown in Figure 3, reducing the number of available training print jobs does not eliminate the benefit of LARM for most models. Although the magnitude of improvement varies across architectures, LARM continues to outperform the baseline for six of the eight classifiers even when the training set is restricted to only nine print jobs. The largest gains are observed for recurrent architectures, with the improvement increasing from 0.093 to 0.201 for LSTM and from 0.058 to 0.192 for GRU.

These results are notable because reducing the number of training jobs also reduces the diversity of information available for augmentation, which could potentially limit the effectiveness of LARM. Nevertheless, LARM retains its performance advantage for most classifiers under the reduced-data setting. The substantially larger gains observed for LSTM and GRU further suggest that LARM remains particularly effective for classifiers that are more susceptible to overfitting, even under severe data scarcity (see Section 3.2.5).

#### 3.2.7. Sensitivity Analysis of Level Mixing and Pairing Strategy

LARM introduces the level-mixing ratio μ, which controls the contribution of the paired sample’s level component to the augmented signal. To examine the sensitivity of LARM to this hyperparameter, we evaluate six level-mixing ratios, μ∈{0.0,0.2,0.4,0.6,0.8,1.0}, for each classification model.

**Figure 4:**
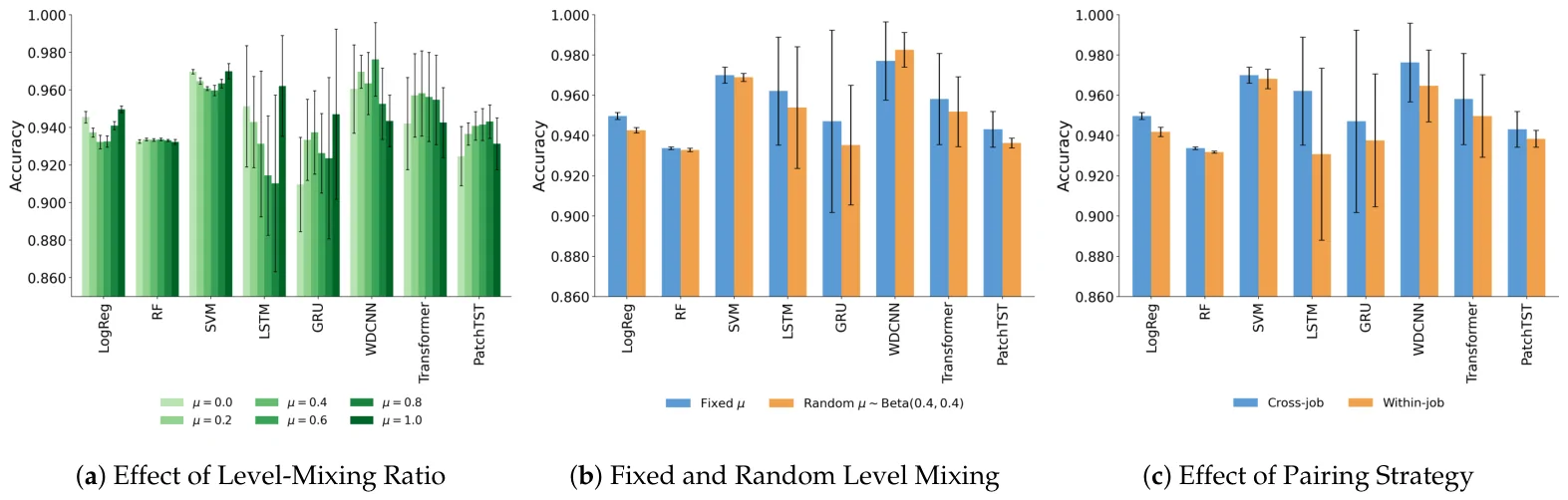
(**a**) Accuracy of LARM under different level-mixing ratios (μ∈{0.0,0.2,0.4,0.6,0.8,1.0}). (**b**) Comparison of LARM performance using the fixed μ and random μ∼Beta(0.4,0.4). (**c**) Comparison of LARM performance under cross-job and within-job pairing for each classification model.

Figure 4a shows that the optimal value of μ varies substantially across models. Logistic regression, SVM, LSTM, and GRU achieve their highest accuracy at μ=1.0, whereas random forest, WDCNN, Transformer, and PatchTST perform best at intermediate values. These results indicate that the optimal level-mixing ratio depends on the classification model and that μ should be tuned per classification model rather than fixed a priori.

To further examine whether randomizing the level-mixing ratio can provide an alternative to selecting a fixed μ, we independently sample μ from Beta(0.4,0.4) for each augmented sample. As shown in Figure 4b, the fixed-μ setting achieved slightly higher accuracy for seven of the eight classifiers, indicating that randomizing μ does not provide a clear performance advantage over explicitly controlling the degree of level mixing.

We next examine the sensitivity of LARM to the pairing strategy. LARM selects the paired sample from the same defect class but a different print job than the anchor sample. This design is motivated by the expectation that residual patterns within the same print job are highly similar, while those from different print jobs may provide greater within-class variation. To evaluate this design choice, we compare the default cross-job strategy with an alternative within-job strategy, in which the paired sample is selected from the same print job as the anchor.

As shown in Figure 4c, cross-job pairing outperforms within-job pairing across all eight classification models, with accuracy improvements ranging from 0.002 to 0.031. The largest improvement is observed for LSTM, while even the smallest improvements remain positive. This result supports the use of cross-job pairing in LARM and indicates that pairing samples from different print jobs is more effective than restricting pairing to samples from the same print job.

## 4. Discussion

The key finding of this study is that the effectiveness of data augmentation in repeated-measures settings depends on how job-specific information is distributed within the signal. The ICC analysis in Section 3.2.1 shows that job-specific characteristics are concentrated primarily in the level component, whereas the residual component contains relatively little such information. This asymmetry has important implications for the design of augmentation strategies. Since the level component captures much of the job-specific information, augmentation methods that modify the entire signal indiscriminately may distort realistic job-level characteristics. In contrast, LARM explicitly accounts for this asymmetry by handling the level and residual components separately, preserving realistic level information while allowing flexible residual mixing. The consistent improvements across all eight classification models suggest that component-aware augmentation is particularly beneficial for improving generalization in repeated-measures settings with a limited number of independent print jobs.

The comparison with standard mixup (see Section 3.2.3) further supports the benefit of treating the level and residual components separately. Standard mixup applies a single interpolation ratio to the original signal, implicitly treating both components identically [20]. In contrast, LARM independently controls the mixing of the level and residual components, allowing realistic level characteristics to be preserved while retaining the flexibility of residual mixing. The component-wise ablation analysis (see Section 3.2.4) also shows that neither level-only nor residual-only mixing consistently outperforms the other across classification models, while LARM achieves the highest accuracy in most cases. These results suggest that explicitly accounting for the distinct roles of these two components through separate mixing control contributes to improved generalization.

In Section 3.2.5, we further clarify when LARM is most effective. Larger baseline train–test gaps are associated with greater performance gains, suggesting that LARM is particularly effective for classifiers that are more prone to overfitting. This trend remains evident even when the number of available training print jobs is substantially reduced (Section 3.2.6), indicating that the benefit of LARM can be maintained under more severe data scarcity. These observations are consistent with the motivation of LARM, which aims to mitigate overfitting arising from the repeated-measures structure.

The sensitivity analysis in Section 3.2.7 also supports the design of LARM. Cross-job pairing consistently outperforms within-job pairing, suggesting that pairing samples from different print jobs provides more effective augmentation than restricting pairing to samples from the same print job.

These findings also have practical implications for the use of LARM in real-time in-situ monitoring during MEX processes. LARM serves as a training-time augmentation method to improve the generalization of classifiers trained on a limited number of independent print jobs. Once the classifier has been trained, it can be deployed for in-situ monitoring to classify incoming sensor segments without additional augmentation. If the classifier is retrained as additional labeled print-job data become available, LARM can be reapplied during the retraining stage. In this way, LARM can support real-time defect classification by improving generalization to unseen print jobs.

Despite its effectiveness, LARM has several limitations. First, the experiments were conducted on a single dataset consisting of relatively slowly varying environmental sensor signals, for which job-specific variations may naturally be concentrated in the level component. Therefore, it remains unclear whether the observed distribution of job-specific information between the level and residual components generalizes to other MEX processes and sensor modalities. In particular, dynamic signals such as vibration, acoustic emission, or motor current may exhibit job-specific variations in spectral or waveform characteristics beyond the level component, potentially requiring more sophisticated decomposition strategies. Second, LARM requires selecting an appropriate level-mixing ratio, whose optimal value varies across classifiers, introducing additional tuning cost. Developing adaptive mechanisms for selecting this parameter would improve the applicability of the proposed method. Finally, LARM selects paired samples randomly within the same defect class. Incorporating signal similarity or print-job relationships into the pairing process may produce more informative augmented samples. In future work, we will evaluate LARM on diverse MEX datasets and further refine its augmentation strategy to improve its generality and applicability.

## 5. Conclusions

To improve generalization in data-driven in-situ defect classification under repeated-measures settings in MEX, we propose a novel data augmentation method, LARM, that separately augments the level and residual components of sensor signals. This design was motivated by our finding that job-specific characteristics are concentrated primarily in the level component, while the residual component contains little such information. Accordingly, LARM preserves realistic level characteristics while allowing flexible mixing of the residual component during augmentation.

Extensive experiments demonstrated that LARM outperformed representative data augmentation methods across diverse classical machine learning and deep learning models for in-situ defect classification. Additional analyses suggest that LARM is particularly beneficial for classifiers that are more prone to overfitting under repeated-measures settings.

This study demonstrates that explicitly accounting for the distinct roles of the level and residual components provides an effective augmentation strategy for repeated-measures datasets. We hope that the proposed component-aware augmentation strategy will encourage future research on augmentation methods that explicitly account for the characteristics of different signal components across a wide range of application domains.

## Figures and Tables

**Table 1:** Implementation settings and computational environment used in the experiments.

Category	Item	Setting
Machine learning	Logistic regression	Maximum iterations: 3000
	Random forest	Number of trees: 300
	SVM	RBF kernel; C=1.0
Deep learning	Optimizer	Adam; learning rate: $1\times 10^{-3}$
	Batch size	128
	Loss function	Cross-entropy
	Training epochs	12
Data augmentation	Jitter	Noise scale: 0.03
	Scaling	Scaling factor: $N(1,0.1^{2})$
	Time warping	Number of knots: 4; warp scale: 0.2
	Cutout	Cutout ratio: 5–15%
	Frequency masking	Mask ratio: 15%
	SMOTE	Number of nearest neighbors: 5; interpolation ratio: U(0,1)
	DBA	Mixing ratio: λ∼Beta(0.4,0.4); downsampled length: 50
	Mixup	Mixing ratio: λ∼Beta(0.4,0.4)
	LARM	Residual mixing ratio: λ∼Beta(0.4,0.4); level-mixing ratio: μ∈{0,0.2,…,1.0}
Computational environment	Hardware	AMD Ryzen 7 9800X3D (8-core CPU); 60 GB RAM; NVIDIA RTX A5000 (24 GB)
	Software	PyTorch 2.5.1; CUDA 12.1

**Table 2:** Comparison of ICC values between the level and residual components.

Channel	Level ICC	Residual ICC
Gas	0.610	0.005
Humidity	0.830	0.000
Pressure	0.998	0.009
Temperature	0.307	0.000
Average	0.686	0.003

**Table 3:** Classification accuracy achieved by each augmentation strategy across eight classification models. The best result for each model is highlighted in boldface. Baseline: training without augmentation; LogReg: logistic regression; RF: random forest; FreqMask: frequency masking; TimeWarp: time warping.

Strategies	Machine Learning Models			Deep Learning Models				
	LogReg	RF	SVM	LSTM	GRU	WDCNN	Transformer	PatchTST
Baseline	0.926 ±0.000	0.930 ±0.000	0.964 ±0.000	0.869 ±0.047	0.889 ±0.028	0.959 ±0.013	0.920 ±0.036	0.930 ±0.012
Jitter	0.932 ±0.000	0.930 ±0.000	0.967 ±0.002	0.882 ±0.046	0.927 ±0.036	0.968 ±0.027	0.941 ±0.015	0.917 ±0.012
Scaling	0.698 ±0.004	0.856 ±0.014	0.727 ±0.005	0.687 ±0.022	0.676 ±0.014	0.788 ±0.014	0.735 ±0.022	0.725 ±0.018
TimeWarp	0.926 ±0.000	0.930 ±0.000	0.967 ±0.001	0.912 ±0.027	0.924 ±0.036	0.971 ±0.020	0.939 ±0.018	0.917 ±0.012
Cutout	0.926 ±0.001	0.930 ±0.000	0.965 ±0.001	0.938 ±0.033	0.937 ±0.020	0.971 ±0.019	0.939 ±0.018	0.920 ±0.011
FreqMask	0.847 ±0.039	0.923 ±0.010	0.876 ±0.033	0.915 ±0.032	0.939 ±0.028	0.897 ±0.044	0.914 ±0.028	0.933 ±0.011
SMOTE	0.928 ±0.002	0.932 ±0.001	0.959 ±0.002	0.921 ±0.031	0.926 ±0.027	0.951 ±0.015	0.944 ±0.019	0.928 ±0.015
DBA	0.928 ±0.003	0.933 ±0.001	0.962 ±0.002	0.910 ±0.047	0.909 ±0.029	0.964 ±0.022	0.956 ±0.019	0.929 ±0.015
Mixup	0.930 ±0.000	0.932 ±0.001	0.965 ±0.003	0.934 ±0.026	0.911 ±0.022	0.962 ±0.022	0.950 ±0.010	0.937 ±0.020
LARM	**0.950** ±0.002 **	**0.934** ±0.001 **	**0.970** ±0.004 *	**0.962** ±0.027 **	**0.947** ±0.045 **	**0.976** ±0.020	**0.958** ±0.023 **	**0.943** ±0.009 *
*p*-value	$1.146\times 10^{-5}$	$4.460\times 10^{-4}$	$5.161\times 10^{-2}$	$2.433\times 10^{-2}$	$4.099\times 10^{-2}$	$1.329\times 10^{-1}$	$9.158\times 10^{-3}$	$9.616\times 10^{-2}$

**Table 4:** Quantitative analysis of level distortion induced by scaling.

Channel	Out-of-Range Fraction	Mean Absolute z-Score
Gas	0.063±0.005	2.908±0.085
Humidity	0.104±0.007	2.730±0.091
Pressure	0.907±0.005	11.332±0.315
Temperature	0.395±0.009	3.746±0.145

**Table 5:** Classification accuracy under different level and residual mixing strategies. The best result for each model is highlighted in boldface.

Model	No Mixing	Level-Only	Residual-Only	Standard Mixup	LARM
LogReg	0.926±0.000	0.945±0.003	0.946±0.003	0.930±0.000	0.950±0.002
RF	0.930±0.000	0.933±0.001	0.933±0.001	0.932±0.001	0.934±0.001
SVM	0.964±0.000	0.971±0.002	0.970±0.001	0.965±0.003	0.970±0.004
LSTM	0.869±0.047	0.938±0.012	0.951±0.032	0.934±0.026	0.962±0.027
GRU	0.889±0.028	0.928±0.006	0.910±0.025	0.911±0.022	0.947±0.045
WDCNN	0.959±0.013	0.960±0.018	0.962±0.023	0.962±0.022	0.977±0.020
Transformer	0.920±0.036	0.956±0.019	0.942±0.025	0.950±0.010	0.958±0.023
PatchTST	0.930±0.012	0.930±0.015	0.925±0.016	0.937±0.020	0.943±0.009

## Data Availability

The original contributions presented in this study are included in the article. Further inquiries can be directed to the corresponding author.
